# Transcervical Radiofrequency Ablation of Symptomatic Uterine Fibroids: 2-Year Results of the SONATA Pivotal Trial

**DOI:** 10.1089/gyn.2019.0012

**Published:** 2019-12-09

**Authors:** Charles E. Miller, Khadra M. Osman

**Affiliations:** ^1^ The Advanced Gynecologic Surgical Institute, Schaumburg, Illinois.; ^2^ Fort Lauderdale Women Care, Ft. Lauderdale, Florida.

**Keywords:** transcervical fibroid ablation, radiofrequency ablation, leiomyoma, uterine fibroids, quality of life, ultrasonography

## Abstract

***Objective:*** To report 2-year results of sonography-guided transcervical fibroid ablation (TFA) using the Sonata^®^ system in women with symptomatic uterine fibroids.

***Design:*** This is a prospective multicenter single-arm interventional trial.

***Methods:*** Premenopausal women with up to 10 clinically relevant uterine fibroids, each ranging from 1 to 5 cm in diameter, were treated with sonography-guided TFA on an outpatient basis and returned for regular follow-up visits for 2 years. Assessed outcomes included changes in symptom severity, heath-related quality of life, general health status, work and activity limitations, treatment satisfaction, adverse events, surgical reintervention, and occurrence of pregnancy and associated outcomes.

***Results:*** Among 147 enrolled women, 125 (85%) returned for follow-up at 2 years. Compared with baseline, symptom severity decreased from 55 ± 19 to 24 ± 18 (*p* < 0.001), health-related quality of life increased from 40 ± 21 to 83 ± 19 (*p* < 0.001), and EuroQol 5-Dimension scores increased from 0.72 ± 0.21 to 0.89 ± 0.14 (*p* < 0.001). Overall treatment satisfaction at 2 years was 94%. The mean percentage of missed work time, overall work impairment, and activity impairment significantly decreased at follow-up. Through 2 years, surgical reintervention for heavy menstrual bleeding was performed in 5.5% of patients. One singleton pregnancy occurred with a normal peripartum outcome.

***Conclusions:*** TFA treatment with the Sonata system provides significant clinical improvement through 2 years postablation, with a low incidence of surgical reintervention. Other favorable outcomes included a rapid return to work and substantial improvements in quality of life, symptom severity, work productivity, and activity levels.

## Introduction

Uterine fibroids are a highly prevalent gynecologic condition and can be identified in at least 70% of women by the age of 50 years.^1^ Many women with fibroids are asymptomatic and require no intervention. However, at least one in three women with fibroids report symptoms such as heavy menstrual bleeding (HMB) and/or bulk symptoms that interfere with activities of daily living.^2^ Women diagnosed with uterine fibroids also have a higher risk of anemia and infertility than women without this diagnosis.^3,4^ Self-management of symptoms with nonprescription medication or lifestyle modification before seeking medical care is common, but often unsuccessful.^5^

Initial management of symptomatic fibroids may be guided by the patient's desire for future fertility. More than 200,000 hysterectomies are performed each year in the United States for the treatment of symptomatic fibroids.^6^ However, there is growing concern that hysterectomy for fibroid treatment is overutilized^7^ and patients are increasingly seeking less invasive uterus-preserving treatment options.^5^ Myomectomy and uterine artery embolization (UAE) are uterus-preserving alternatives to hysterectomy that may be appropriate for well-selected patients. However, the acceptability of these treatments may be limited since 79% of women with symptomatic fibroids desire treatments that do not involve invasive surgery and 65% of women younger than 40 years prefer a treatment that preserves fertility.^5^ In the case of UAE, future pregnancy is not recommended, and successful pregnancy outcomes are reduced after such treatment. Surgical reintervention rates for hysterectomy alternatives have been reported as high as 23.5% at 2 years.^8–11^ Given the lack of treatment options that align with these preferences, women with symptomatic fibroids represent an underserved population who would benefit from the development of safer, less invasive, and equally or more effective treatment options.

Use of radiofrequency (RF) ablation as a therapeutic option for solid tumors has been increasing over the past two decades among various therapeutic areas. RF ablation heats targeted tissue, causing coagulative necrosis. To better address the needs of women with symptomatic fibroids, an incisionless uterus-preserving sonography-guided transcervical fibroid ablation (TFA) outpatient procedure has been developed. In the sonography-guided transcervical ablation of uterine fibroids (SONATA) pivotal trial, performed under an investigational device exemption (IDE) from the U.S. Food and Drug Administration (FDA), clinically meaningful improvements in patient-reported symptoms, no device-related complications, and a surgical reintervention rate of <1% were reported through 1 year.^12^ To characterize longer term safety and efficacy results with this procedure, we present 2-year results from this pivotal trial of sonography-guided transcervical RF ablation in women with symptomatic uterine fibroids.

## Materials and Methods

### Study design

SONATA was a prospective multicenter single-arm interventional trial of sonography-guided TFA in women with symptomatic uterine fibroids. The clinical trial was performed under an IDE approved by the FDA in the United States and the Federal Commission for Protection against Health Risks (COFEPRIS) in Mexico. Study enrollment began in April of 2015 and ended in October of 2016. Each patient provided informed consent to participate in the trial, and every clinical site obtained local institutional review board or ethics committee approval before commencing patient enrollment. The study was registered at ClinicalTrials.gov (NCT02228174).

### Participants

Eligible subjects were premenopausal women aged 25 to 50 years with regular and predictable menstrual cycles, objective evidence of HMB, and with up to 10 fibroids of International Federation of Gynecology and Obstetrics (FIGO) types 1, 2, 3, 4, and/or 2–5 (transmural), each from 1 to 5 cm diameter. Types 5 and 6 subserous myomata were not counted in the total number of fibroids but could be ablated at the discretion of the investigator. At least one fibroid was required to have either indented or abutted the endometrial cavity (FIGO type 1, type 2, type 3, or types 2–5 fibroids). Women were excluded if they expressed a desire for future pregnancy, had any type 0 fibroids ≥1.0 cm or endometrial polyps ≥1.5 cm or multiple polyps of any size, bulk symptoms attributable to subserous fibroids, prior confounding procedures (endometrial ablation, UAE, uterine artery occlusion, or hyperthermic ablation of fibroids), uterine volume ≥1000 cm^3^, presence of tubal implants for sterilization, and/or clinically significant adenomyosis.

### Procedure

Clinical sites with community or academic gynecologists with generalist experience in hysteroscopic and/or laparoscopic surgery participated in the trial. Gynecologist training for the procedure entailed didactic instruction and practice on physical uterine models with various fibroid sizes, types, and locations. The sonography-guided TFA procedure used in the trial has been described in detail elsewhere.^12^ The treatment device (Sonata^®^ system; Gynesonics, Inc., Redwood City, CA) consists of an integrated intrauterine sonography probe and RF ablation handpiece that allows the gynecologist to identify, target, and ablate uterine fibroids. The integration of real-time ultrasound imaging enables the physician to visualize, target, and ablate a greater range of fibroids than could be approached through operative hysteroscopy.^12^ A graphical interface is displayed on the live ultrasound image that identifies the target ablation area and the extent of subablative thermal heating. The gynecologist utilizes this information to confirm the ablation is within the fibroid while confining the thermal safety border to within the uterine serosa. A single ablation may suffice to treat a fibroid, but additional ablations may be necessary depending on fibroid size, location, and geometry. Anesthesia was individualized; general anesthesia was not a requirement.

### Follow-up and outcomes

Patients returned for follow-up visits at 10 days, 30 days, 3 months, 6 months, 1 year, and 2 years. Follow-up remains ongoing in this trial through 3 years. Outcomes at 2 years included changes in symptom severity, health-related quality of life (HRQL), general health, and work/activity limitations; serious adverse events; surgical reintervention for HMB; and occurrence of pregnancy and associated outcomes. Symptom severity and quality of life were assessed with the symptom severity score (SSS) and HRQL subscales of the uterine fibroid symptom and quality-of-life questionnaire.^13^ Scores are reported on a 0 to 100 scale where higher SSSs indicate more severe symptoms and lower HRQL scores indicate worse quality of life. Changes in general health status were assessed with the EuroQol 5-Dimension (EQ-5D) questionnaire. The EQ-5D consists of five questions that provide a description of the patient's health state with scores ranging from 0 (indicating death) to 1 (indicating perfect health). Patients self-reported their perceived treatment benefit at 2 years as *improved*, *no change*, or *worsened*. Treatment satisfaction was measured on a 6-item scale ranging from *very satisfied* to *very dissatisfied*. The work productivity and activity impairment questionnaire: specific health problem questionnaire^14^ assessed change in work and activity patterns after treatment. Overall patient treatment outcome was assessed using the overall treatment effect (OTE) scale. Adverse events were reported according to seriousness and relationship with the device or procedure.

### Statistical analysis

Safety analyses included all treated patients and efficacy analyses excluded patients who reached menopause during follow-up. Data were reported using the mean and standard deviation for normally distributed continuous outcomes, median and interquartile range for non-normally distributed continuous data, and count and frequency for categorical data. The Wilcoxon signed-rank test assessed change over time for symptom severity, HRQL, general health, and work/activity limitation outcomes. Reintervention due to HMB was analyzed using Life-Table methods, with a sensitivity analysis using binomial methods (i.e., event count divided by evaluable sample size). Data were analyzed using SAS version 9.3 (SAS Institute, Cary, NC). All statistical tests were two sided, and *p*-values of <0.05 indicated statistical significance.

## Results

A total of 147 women (mean age 43 years, body mass index 29 kg/m^2^) were enrolled at 22 sites (21 in the United States, 1 in Mexico). Demographic characteristics of the patients are provided in Table 1. All patients presented with HMB and their general health status measured on the EQ-5D was below the 25th percentile compared with sex- and age-matched population norms.^15^ A mean of 3.0 (±2.1) fibroids per patient were treated with transcervical RF ablation. Patient characteristics and procedural details have been previously reported.^12^ A total of 125 (85%) patients returned for follow-up at 2 years. Six patients missed the 2-year follow-up visit and 16 patients withdrew from the study before the 2-year follow-up visit (none due to an adverse event).

**Table 1.** Baseline Patient Characteristics

Variable	Value (*n* = 147)
Age, years	42.9 ± 4.3 [31, 50]
Ethnicity	
Hispanic or Latina	43 (29.3)
Not Hispanic or Latina	104 (70.7)
Race^a^	
American Indian or Alaska Native	3 (2.0)
Asian	2 (1.4)
Black or African American	49 (33.3)
Native Hawaiian or other Pacific	1 (0.7)
White	60 (40.8)
Other	33 (22.4)

Values are mean ± standard deviation [minimum, maximum], or count (percentage).

^a^ Patients can be counted more than once if multiple races were indicated.

**FIG. 1.**
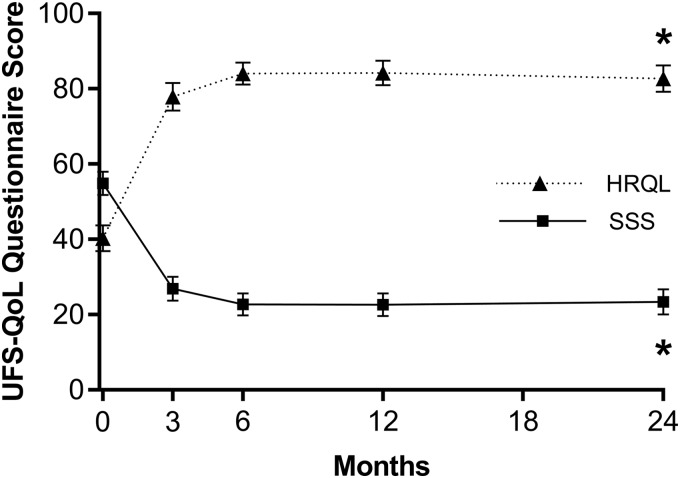
Change in SSS and HRQL subscales of the UFS-QoL questionnaire for 2 years after sonography-guided transcervical fibroid ablation. Lower SSSs indicate less severe symptoms. Higher HRQL scores indicate better quality of life. Plotted values are mean and 95% confidence interval. *p < 0.001 for change from baseline. HRQL, health-related quality of life; SSS, symptom severity score; UFS-QoL, uterine fibroid symptom and quality of life.

**FIG. 2.**
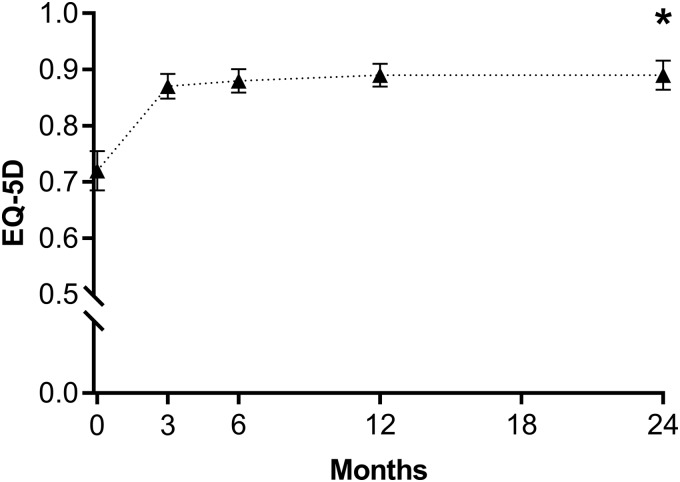
Change in EQ-5D questionnaire score for 2 years after sonography-guided transcervical fibroid ablation. Plotted values are mean and 95% confidence interval. *p < 0.001 for change from baseline. EQ-5D, EuroQol 5-Dimension.

Over the 2-year follow-up period, mean values on the SSS decreased from 55 ± 19 to 24 ± 18 (*p* < 0.001), HRQL scores increased from 40 ± 21 to 83 ± 19 (*p* < 0.001) (Fig. 1), and EQ-5D scores increased from 0.72 ± 0.21 to 0.89 ± 0.14 (*p* < 0.001) (Fig. 2). Patient satisfaction with treatment at 2 years was 94% (75% of patients reported they were very satisfied, 13% were moderately satisfied, 6% were somewhat satisfied, 0% were somewhat dissatisfied, 4% were moderately dissatisfied, and 2% were very dissatisfied). At 2 years, 88% of patients reported improvement in fibroid symptoms on the OTE questionnaire compared with baseline. Indicators of work impairment due to fibroid symptoms significantly improved from baseline to 2 years. The percentage of missed work time significantly decreased from a mean of 2.9% to 1.3% (*p* < 0.001) and the overall percentage of work impairment also demonstrated significant improvement, being reduced from a mean of 51% to 14% (*p* < 0.001). Patients also reported significant reductions in the percentage of activity impairment due to fibroid symptoms over this period (mean of 58% to 14%, *p* < 0.001).

One-year safety outcomes with sonography-guided transcervical RF ablation in this trial were previously reported.^12^ In brief, procedure-related serious adverse events were reported in 2 (1.4%) patients during the first year of follow-up without any device-related adverse events during this period. Between the 1- and 2-year follow-up visits, there were no serious adverse events and no adverse events related to the device or procedure. The cumulative rate of surgical reintervention for HMB through 2 years was 5.2% (95% confidence interval [CI]: 2.5%–10.6%) using Life-Table methods and 5.5% (95% CI: 2.2%–11.0%) using binomial methods. One singleton pregnancy was reported in a 36-year-old multigravida who conceived 22 months after ablation. The patient delivered a liveborn male infant at 38 2/7 weeks gestation by elective repeat cesarean section with Apgar scores of 9^1^/10^5^ and a birth weight of 4005 g. Visual inspection of the endometrial cavity appeared within normal limits and there was no evidence of uterine dehiscence or rupture.

## Discussion

Women with symptomatic uterine fibroids are often treated with hysterectomy and other significantly invasive and potentially morbid procedures. Although hysterectomy is definitive treatment for fibroids, it removes the possibility of future pregnancy, and there are concerns about potential need for blood products; complications (including long-term effects such as pelvic prolapse), lost work time; possible earlier menopause; and increased osteoporosis risk.^5,16,17^

Most affected women prefer uterine-conserving treatments irrespective of their desire for childbearing.^5^ Therefore, a safe, effective, and convenient less invasive treatment option for symptomatic fibroids would be welcomed by patients and health care providers alike. Sonography-guided TFA was developed to bridge this therapeutic gap. Although promising results with this technology have been reported in previous studies,^18,19^ this study is the largest conducted to date. The 2-year results of the SONATA Pivotal IDE Trial demonstrate that sonography-guided TFA is a safe outpatient incisionless and uterus-preserving procedure, which provides significant durable symptom relief, and significantly improves general and condition-specific quality of life through 2 years.

Results with sonography-guided TFA in the current trial are comparable with those reported in previously published studies with this technology. A clinical trial (Fibroid Ablation Study-EU) in Europe and Mexico that followed 50 patients treated with the Sonata System (previously VizAblate^®^) for 1-year reported encouraging results.^19^ During follow-up, fibroid volume decreased by 67%, fibroid symptoms significantly improved, and overall safety was excellent. Long-term results from the VITALITY Clinical Study demonstrated that symptom resolution persisted in 17 women treated with the Sonata System for 5.4 years mean follow-up, with no surgical reintervention for the first 3.5 years, a 11.8% rate of surgical reintervention at 5 years for HMB, and an overall surgical reintervention event rate of 2.2% per year.^20^ One-year results from the current SONATA trial were previously reported in which fibroid volume decreased by 62%, fibroid symptoms significantly improved, and the surgical reintervention rate was 0.7%. The current report extends these previous results to 2 years, in which efficacy was durably maintained, one pregnancy with a normal peripartum outcome resulted, and no new safety concerns were identified. The 2-year surgical reintervention rate reported herein for TFA of uterine fibroids was 5.5%. This is noteworthy, considering the rates of reintervention for other procedures reported at 2 years were 23.5% for UAE, 18% for hysteroscopic myomectomy, 19% for endometrial ablation, and 8% for laparoscopic myomectomy.^9,21^

TFA was performed with acceptable safety in this study. Transcervical access involves no incisions and avoids the peritoneal cavity, which minimizes iatrogenic complication risks inherent with transperitoneal surgery such as wound infection or ureteral injury. Patients were discharged ∼2 hours after the procedure and treatment satisfaction was high. Although general anesthesia was used in 50% of patients largely due to patient or anesthesiologist preference, it is not a requirement and most patients treated with conscious sedation experienced minimal procedural discomfort. Because a varied mixture of community and academic generalist gynecologists were recruited and provided treatment after a brief standardized didactic and practical training program, this procedure appears to be generalizable to a broad range of gynecologists since specialized sonography expertise was neither required nor assumed. As with any new treatment, thoughtful patient selection and careful attention to the prescribed procedural technique are important for optimal results. Thorough differential diagnosis should be performed to determine whether patient symptoms are attributable to uterine fibroids or other causes such as anovulation, adenomyosis, or bleeding disorders. Shared decision-making between patient and provider should dictate the preferred procedure for symptomatic fibroid treatment. Published clinical results demonstrate that TFA is a safe and effective option that can be included in the gynecologist's armamentarium of treatment options among patients seeking treatment for symptomatic fibroids.

## Conclusion

TFA treatment with the Sonata system provides significant clinical improvement through 2 years postablation, with a low incidence of surgical reintervention and a favorable safety profile. Other outcomes included a rapid return to work and substantial improvements in quality of life, symptom severity, work productivity, and activity levels.
